# VQGNet: An Unsupervised Defect Detection Approach for Complex Textured Steel Surfaces

**DOI:** 10.3390/s24196252

**Published:** 2024-09-27

**Authors:** Ronghao Yu, Yun Liu, Rui Yang, Yingna Wu

**Affiliations:** Center for Adaptive System Engineering, ShanghaiTech University, Shanghai 201210, China; yurh2022@shanghaitech.edu.cn (R.Y.); liuyun2023@shanghaitech.edu.cn (Y.L.); yangrui@shanghaitech.edu.cn (R.Y.)

**Keywords:** defect detection, unsupervised anomaly algorithm, VQGNet, feature fusion

## Abstract

Defect detection on steel surfaces with complex textures is a critical and challenging task in the industry. The limited number of defect samples and the complexity of the annotation process pose significant challenges. Moreover, performing defect segmentation based on accurate identification further increases the task’s difficulty. To address this issue, we propose VQGNet, an unsupervised algorithm that can precisely recognize and segment defects simultaneously. A feature fusion method based on aggregated attention and a classification-aided module is proposed to segment defects by integrating different features in the original images and the anomaly maps, which direct the attention to the anomalous information instead of the irregular complex texture. The anomaly maps are generated more confidently using strategies for multi-scale feature fusion and neighbor feature aggregation. Moreover, an anomaly generation method suitable for grayscale images is introduced to facilitate the model’s learning on the anomalous samples. The refined anomaly maps and fused features are both input into the classification-aided module for the final classification and segmentation. VQGNet achieves state-of-the-art (SOTA) performance on the industrial steel dataset, with an I-AUROC of 99.6%, I-F1 of 98.8%, P-AUROC of 97.0%, and P-F1 of 80.3%. Additionally, ViT-Query demonstrates robust generalization capabilities in generating anomaly maps based on the Kolektor Surface-Defect Dataset 2.

## 1. Introduction

Defect detection is crucial and indispensable in the industrial manufacturing process. Presently, professionally trained inspectors primarily conduct product defect detection. However, manual inspection is susceptible to subjective biases, low efficiency, lengthy training periods, and high labor costs. With the rapid advancements in computer vision and industrial automation, employing computer vision to replace manual inspection is increasingly becoming the mainstream strategy for industrial defect detection. Notably, high-accuracy defect detection algorithms are essential for the automatic identification of defects. Currently, defect detection methods can be categorized into two types, which are traditional descriptor-based [1,2] and deep learning-based methods [3,4,5,6,7,8,9,10], respectively. Traditional methods typically rely on handcrafted features, including texture-based, shape-based, and color-based detection methods [11,12]. These methods do not require large datasets to learn features, while their adaptability is relatively lower due to the lack of expert knowledge compared with deep learning-based methods. Although requiring large datasets for training, deep learning algorithms deliver powerful feature extraction capabilities by different trained models. According to the tasks of defect detection, deep learning-based methods are classified into supervised, semi-supervised, and unsupervised methods [13]. At present, the detection of surface defects in steel frequently involves manually annotated anchor boxes, employing supervised object detection algorithms to concurrently accomplish defect detection, classification, and localization tasks [14,15,16]. However, in cases where defect classification is unnecessary and only defect detection and localization are required, supervised object detection algorithms necessitate a significantly substantial workload. Although object detection algorithms exhibit remarkable performance in defect localization compared to image segmentation algorithms, they are often inferior to the latter in segmenting the defect regions. Since unsupervised anomaly detection algorithms can perform defect identification and localization tasks using only normal samples, unsupervised anomaly detection algorithms also hold a significant place in the field of defect detection on the surface of steel. However, it is still a challenge for unsupervised algorithms to precisely localize and segment defects with complex backgrounds due to the lack of prior knowledge about defect samples, such as shape, color, size, and semantic information. In current industrial manufacturing, the surface of steel presents complex textures for decorative purposes and corrosion prevention, such as spangle texture on the galvanized steel, which varies in size and often exhibits irregular shapes [17]. Although texture recognition has been investigated for these kinds of steel surfaces [17], studies of defect detection in such textures have not yet been reported. There are two main challenges to precisely recognizing and segmenting defects in this kind of steel: **(1) Complex texture background**: The size of the texture on steel surfaces is variable and not uniform, which results in complex texture information and obstructs the recognition and accurate positioning of defects, especially tiny defects. Moreover, the complex texture also induces a time-consuming annotation process. **(2) illumination variation**: Illumination variation, such as overexposed and underexposed conditions, significantly affects the recognition of defects.To address these challenges, we propose VQGNet (ViT-Query with GalvaNet), an unsupervised algorithm that can precisely recognize and segment defects simultaneously. A feature fusion method, GalvaNet, based on an aggregated attention and a classification-aided module is proposed to segment defects by integrating different features in the original images and anomaly maps. In the aggregated attention module, we designed three components: the image weighting scheme (IWS), multi-scale biaxial cross-attention (MBCA), and coordinate spatial attention (CSA). The IWS aims to dynamically weight the input anomaly map and original image; MBCA is designed to enhance the selection of multi-scale features under complex textures; and CSA strengthens the model’s ability to extract the coordinate and spatial information of features. GalvaNet can generate the refined anomaly map through learning from the original image, including defect-free and defective images with the original size and anomaly maps during training. Moreover, the anomaly maps are generated more confidently via ViT-Query, a method based on vision transformer (ViT) [18] trained on text–image matching tasks, multi-scale feature fusion, and neighbor feature aggregation. An improved synthetic anomaly generation method suitable for grayscale images is also introduced to facilitate the model’s learning on anomalous samples. In summary, our main contributions are as follows:

1. We propose VQGNet, an unsupervised defect detection approach on complex textured surfaces of steel.
2. A feature fusion method named GalvaNet is proposed, which can improve the performance of segmentation of complex textured steel surfaces.
3. We design three components: the image weighting scheme (IWS), multi-scale biaxial cross-attention (MBCA), and coordinate-spatial attention (CSA). The IWS aims to dynamically weight the input anomaly map and original image; MBCA is designed to enhance the selection of multi-scale features under complex textures; and CSA strengthens the model’s ability to extract the coordinate and spatial information of features.
4. An anomaly detection algorithm based on a ViT backbone named ViT-Query, which is enriched with semantic information, multi-feature fusion, and neighbor feature aggregation methods, is proposed as an encoder to generate more confident anomaly maps.
5. A method for creating synthetic anomaly images from grayscale images is also proposed, enabling the rapid and cost-effective generation of a large number of synthetic defects on spangled surfaces.

## 2. Related Work

### 2.1. Unsupervised Anomaly Detection

Unsupervised anomaly detection (UAD) is widely applied as a method of out-of-distribution detection, categorized into three main types: reconstruction-based, flow-based, and embedding-based methods. Reconstruction-based methods are commonly used for anomaly detection in images and video surveillance. Generative models, such as autoencoders (AEs) [19] and generative adversarial networks (GANs) [20], are designed to identify anomalies by comparing the deviations between reconstructed normal and anomalous data. DRÆM [21] features a dual-subnetwork architecture with the reconstruction subnetwork trained to detect and reconstruct semantically plausible anomalies and preserve non-anomalous regions. Liu et al. [22] employed a straightforward architecture comprising a feature extractor, adapter, anomaly feature generator, and discriminator. To improve the model’s generalization, they processed extracted image features, combined them with artificially generated anomalous maps using Gaussian noise, and fused them to produce final anomalous feature maps. Secondly, flow-based methods are another method for unsupervised anomaly detection, normalizing flows to assess data density and assign low likelihoods to anomalies. Rudolph et al. [23] proposed Differnet to improve the performance of models by normalizing flows at multiple scales to capture size variations of defects. Gudovskiy et al. [24] and Rudolph et al. [25] further develop Differnet by constructing multi-scale feature pyramids and introducing fully convolutional cross-scale flow modules, which optimize information exchange and expand receptive fields for precise anomaly localization. Thirdly, embedding-based methods construct a reference gallery of typical data representations and identify anomalies by contrasting them against this established reference. Initially, deep neural networks are used to derive data representations via SVM classification [26]. Recently, the investigation focused on leveraging pre-trained feature extractors from expansive datasets, aiming to pinpoint distinctive features more effectively. Various methodologies, such as k-nearest neighbors (KNN) clustering, memory banks, and bag-of-features, are utilized to assemble these features. PatchCore [27] implements a KNN algorithm on patch-level features to improve efficiency and reduce the potential for anomaly generalization prior to constructing the memory bank. Based on the foundational aspects of PatchCore, PNI [28] integrates comprehensive contextual and positional data for better recognition performance and couples them with a refinement module that follows the initial anomaly map generation to improve the accuracy of segmentation. MemSeg [29] incorporates feature information from normal images into an improved U-Net architecture to enhance the model’s detection and segmentation performance. Although unsupervised anomaly detection has achieved outstanding performance in defect recognition tasks, its performance in defect segmentation and localization tasks is still unsatisfactory, especially for images with complex backgrounds.

### 2.2. Anomaly Simulation-Based Methods

To enhance the segmentation ability of unsupervised models, the generation of artificial anomalies during training has been attempted recently. Li et al. [30] and Song et al. [31] employed a “copy and paste” strategy that involves small rectangular sections of an image that are randomly duplicated and reinserted to simulate structural defects with varying sizes, aspect ratios, and orientations to introduce structural discrepancies. AnomalyDiffusion [32], a few-shot learning method, utilizes diffusion models to learn the characteristics of a small number of anomaly samples and generate more realistic anomaly samples with pixel-level annotations. However, these methods primarily focus on creating either structural or textural anomalies, which may not be effective for datasets with complex backgrounds.

### 2.3. Attention Mechanism

Attention mechanisms are often used to enhance the accuracy of supervised semantic segmentation tasks. To improve the defect segmentation capability of unsupervised anomaly detection algorithms in complex textured backgrounds, introducing attention mechanisms seems to be a good choice. Attention mechanisms were first introduced by Xu et al. [33] for computer vision in 2015. They proposed a novel theory of visual attention that significantly enriched the research landscape. Subsequently, attention mechanisms were developed by Hu et al. [34]. They proposed a spatial attention that dynamically regulates pixel-level weights across feature maps by adjusting the weights of each feature channel. A convolutional block attention module (CBAM) [35] and spatial, channel squeeze, and excitation (SCSE) [34] have been developed to merge spatial and channel attention and introduce additional information for further optimizing the performance. Coordinate attention (CA) [36] captures the cross-channel information and incorporates direction-aware and position-sensitive details, enabling the model to more accurately locate the target areas. EMA [37] introduces a new branch based on coordinate attention to perform 3 × 3 convolution and cross-attention structures to improve the performance of the original attention mechanism. MCA [38] performs strip convolutions on the *x*-axis and *y*-axis, respectively, then computes multi-head cross-attention on the obtained feature maps. Current attention mechanisms are often designed for single attributes, with few combining multiple attributes and being applicable to complex textured surfaces.

## 3. Proposed Method

**Figure 1:**
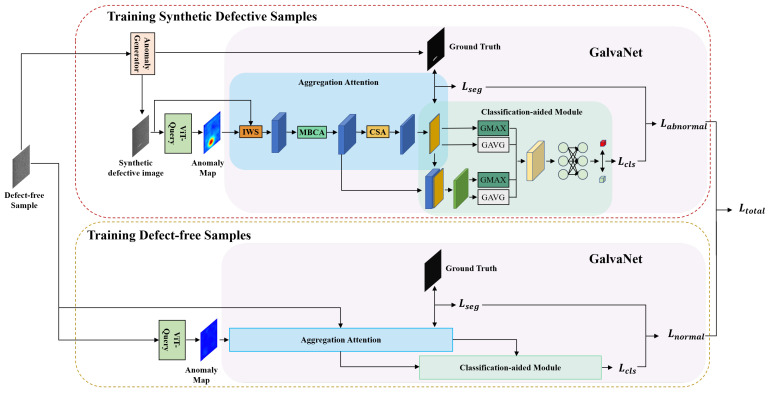
Training process of VQGNet.

**Figure 2:**
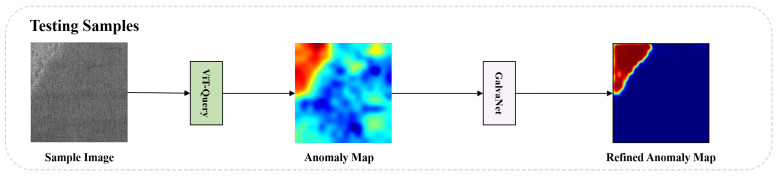
Testing process of VQGNet.

In this section, we present our new framework for recognizing and segmenting defects, VQGNet. The training and testing process is illustrated in Figure 1 and Figure 2, respectively. VQGNet comprises several fundamental components, which we will delineate in the subsequent sections: the anomaly map generation module ViT-Query (Section 3.1), an anomaly generator (Section 3.2), the main multi-task network architecture GalvaNet (Section 3.3), and the loss function (Section 3.4).

### 3.1. ViT-Query

**Figure 3:**
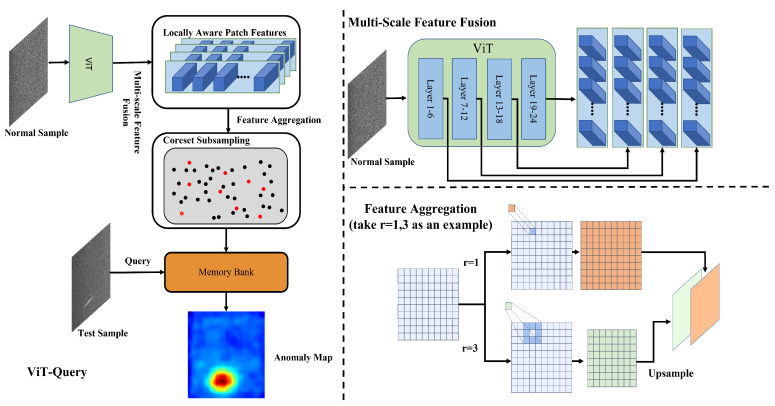
Framework of ViT-Query.

**Feature extraction:** Compared to PatchCore’s utility of WideResNet50 [39] pre-trained on ImageNet [40], ViT-Query utilizes ViT trained on text–image matching tasks to extract features from normal input images. The model will have the ability to discriminate more high-level semantic information after being trained on the task. We divide the ViT into 4 stages, with each stage comprising six layers. This structuring allows for more specialized processing and feature extraction at different levels of the transformer, enhancing the model’s ability to capture and utilize hierarchical semantic information effectively. And, we concatenate the features from every stage, as shown in Figure 3. *I* is the normal image, $\alpha_{i}$ represents the feature extracted by stage *i*, and *L* is the list of features of the image.The process can be represented as follows:

(1) $$\alpha_{i}=f_{stage_{i}}\left(I\right),$$

(2) $$L=Concat(\alpha_{1-4}).$$

**Neighbor feature aggregation:** Using a patch token with multiple aggregation degrees instead of a single patch size is more effective in representing anomalies of varying sizes. This approach results in high-quality anomaly scores. Inspired by Li et al. [41], unlike PatchCore’s directly utilization of 3×3 kernel aggregation, for *i* th patch token $P_{i,j}$ extracted by ViT’s chosen stage *j*, we utilize a r×r kernel to obtain the aggregated neighborhood patch token $\alpha_{agg,r}$ through the $f_{agg,r}$ function and upsample the aggregated patch token into the same size as the initial, as shown in Figure 3. $c_{initial}$, $h_{initial}$ and $w_{initial}$ are the channel height and width of the initial features, respectively. Then, we concatenate the aggregated patch token. $L_{agg}$ is the list of aggregated features. The process can be represented as follows:

(3) $$\alpha_{agg,r}=f_{agg,r}\left(P_{i,j}\right),$$

(4) $$L_{agg}=Concat\left(\left[upsample\left(feature_{agg,r},\left[h_{initial},w_{initial}\right]\right)\right]\right).$$

**Coreset sampling:** When the feature map size and number increase, the memory bank requirements expand, and the inference time rises significantly. ViT-Query addresses this issue by employing greedy coreset subsampling, which reduces the quantity of features. Coreset subsampling produces a subset $M_{coreset}\subseteq L_{agg}$ by utilizing the iterative greedy approximation [42].

**Anomaly Detection:** With the downsampled patch level feature memory bank $M_{coreset}$, ViT-Query queries the anomaly score *s* via the maximum distance $s^{*}$ between input patch features in its patch collection $P_{collection}$ and its nearest neighbor $m^{*}$ in $M_{corset}$:

(5) $$m^{test,*},m^{*}=\underset{m^{test}\in P_{collection}}{\arg\max}\underset{m\in M_{coreset}}{\arg\min}\left|\right|m^{test}-m{\left|\right|}_{2},$$

(6) $$s^{*}=\left|\right|m^{test,*}-m^{*}\left|\right|.$$

During the testing process, there may be rare occurrences where the normal features $m^{*}$ in memory bank $M_{coreset}$ are close to the test features $m^{test,*}$, but these normal features have a low similarity to other normal features. Therefore, the anomaly score $s^{*}$ needs to be adjusted using the following Formula (7) proposed by PatchCore [27]:

(7) $$s=\left(1-\frac{\exp{∥m^{\mathrm{test}\;,*}-m^{*}∥}_{2}}{\sum_{m\in N_{k}\left(m^{*}\right)}\exp{∥m^{\mathrm{test}\;,*}-m∥}_{2}}\right)\cdot s^{*}.$$

$N_{k}$ is the b nearest patch-features in $M_{coreset}$ for test patch-feature $m^{*}$.

### 3.2. Anomaly Generator

**Figure 4:**
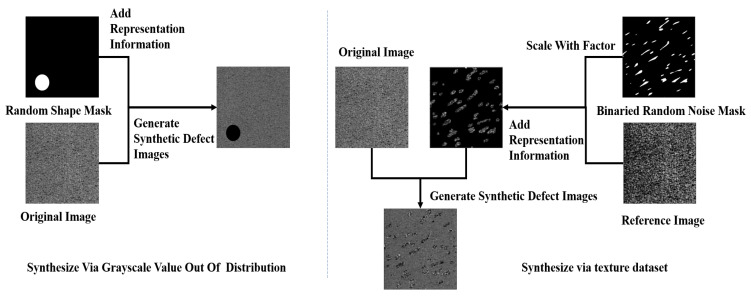
Defective image synthesis strategy.

On the surfaces of steel with complex textures, defects occur in various forms. There are usually small areas of irregularity and large areas of regular defects on the surfaces, and there is a problem of limited and impossible coverage of all defects during the process of data collection, which significantly limits the use of supervised learning methods for modeling. We have designed a more effective strategy, an anomaly generator, to composite defective samples and introduce them during the training process. These synthetic defective samples, based on the principle of random noise and random shape generation, exhibit randomness in the specific locations of defects. The anomaly synthesis strategy proposed consists of three steps: generate a mask, add representation information, and generate synthetic defect images, as shown in Figure 4.

**Generate mask:** In creating anomalies, we start by generating two-dimensional Perlin noise [43], which is then binarized using a threshold T to produce a binary mask. This mask contains random peaks and valleys, which allows model to extract the features of continuous regions or blocks within the image. To composite small-scale irregular defects on the surfaces of steel, further processing is applied to the binarized mask Perlin noise. Multiple connected domain modules within the mask are retained and ensure that each preserved region block has a minimum area of $S_{min}$. These regions are also randomly located due to the randomness of noise. The regions will be scaled by a random scale factor to alter the size of the regions. These preserved regions serve as labels for forged defects, effectively mimicking the small-scale irregularities commonly encountered in industrial environments. For regular defects, a different approach is needed. We generate binary masks with a regular-shaped area, as shown in Figure 4. The masks generated during this process are defined as $M_{initial}$.

**Add representation information:** In the process of generating the mask image $M_{p}$ with the defect information, two methods are introduced to add the defect information, filling the $M_{initial}$ with value $V_{c}$, which is out of the distribution. The reference images $I_{ref}$ form the texture dataset DTD [44]. We assume that the grayscale of a normal image of steel with complex textures conforms to a Gaussian distribution. The value $V_{c}$ is calculated as follows:

(8) $$V_{c}\in \left[0,255\right],$$

(9) p<0.5,

(10) $$V_{c}∼U\left(0,\mu +\Phi^{-1}\left(p\right)\cdot \sigma \right)\;or$$

(11) $$V_{c}∼U\left(\mu -\Phi^{-1}\left(p\right)\cdot \sigma ,255\right).$$

μ and ρ are the mean and variance of the normal imput image, respectively. I(x,y) is the function used to obtain the grayscale value of corrdinate (x,y). mean and var are the functions used to calculate the mean and variance of the image’s grayscale value, respectively. $\Phi^{-1}\left(p\right)$ is the value of the inverse function of the standard normal distribution function at *p*. U(a,b) is a uniform distribution function. The $M_{p}$ in this method is as follows:

(12) $$M_{p}=M_{initiall}⊙V_{c}.$$

⊙ is the element-wise multiply operation. For the method utilizing the $I_{ref}$ value to add the information of defects, image $\beta_{i}\in I_{ref}$ is a random image from the chosen images. $M_{p}$ is expressed as follows:

(13) $$M_{p}=M_{initial}⊙\beta_{i}.$$

**Generate synthetic defect images:** This step inverts the binarized mask $M_{initial}$ to ${\bar{M}}_{initiall}$. After that, it computes the element product on ${\bar{M}}_{initial}$ and the original image *I* to obtain the image $I^{\prime }$ and obtain the augmented image $I_{A}$. The process is based on the following formula:

(14) $$I^{\prime }={\bar{M}}_{initial}⊙I.$$

(15) $$I_{A}={\bar{M}}_{initial}⊙I+I_{n}^{\prime }.$$

**Figure 5:**
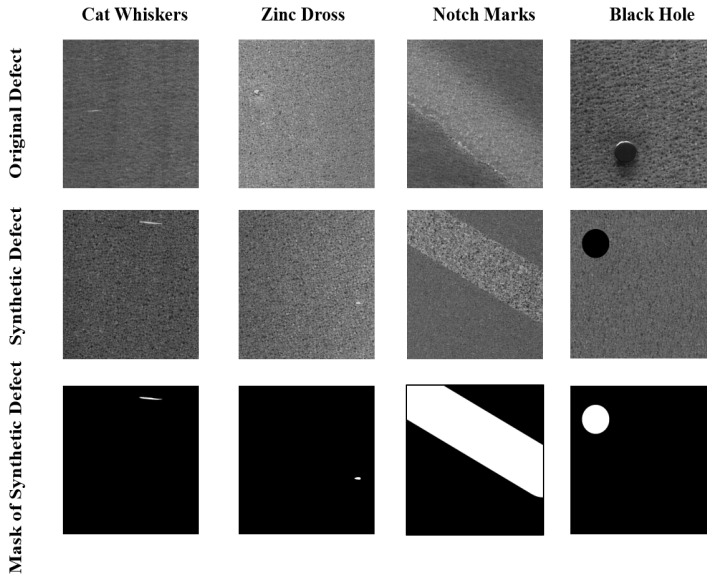
Examples of synthetic defect images.

Using the above strategy, we obtain synthetic anomaly images from the perspectives of texture and structure. Due to the randomness of defect types and positions on steel with complex textures, our synthetic defects will appear anywhere in the image, maximizing the similarity between synthetic anomaly samples and real anomaly samples. Compared to RGB images, which have $255^{3}$ colors, grayscale images only have 255 grayscale values. Owing to this characteristic of grayscale images, when we use out-of-distribution grayscale values to fill the image and create defects, the resulting images are more likely to resemble real defect images, as shown in Figure 5.

### 3.3. GalvaNet

In this section, we introduce a novel network architecture, GalvaNet, which consists of two stages, aggregation attention and the classification-aided module, as shown in Figure 1. This model utilizes synthetic defective images and anomaly maps derived from ViT-Query as network inputs. The purpose of GalvaNet is to leverage the valuable global information from the ViT-Query and enhance the accuracy of defect segmentation and localization. Essentially, this approach aims to augment the model’s capacity to acquire local information while performing segmentation tasks [45].

**Image weighting scheme (IWS):** During the aggregated attention stage, GalvaNet adopts the synthetic defective images and anomaly maps obtained from the ViT-Query as its input. It is cumbersome and inefficient work to set the weight ratio of two inputs manually. To address this issue, GalvaNet employs a channel attention mechanism to integrate input images with anomaly maps, as opposed to utilizing it to re-weight features. It operates by learning the importance of each channel in the input feature map. By dynamically adjusting channel weights, the model can effectively focus on task-relevant information while suppressing irrelevant or noisy channels [46].

In the context of the described mechanism, the input feature maps $X_{\mathrm{ori}}$ and the anomaly maps $X_{\mathrm{ano}}$ obtained from ViT-Query serve as the basis for channel attention. The channel attention mechanism integrates these inputs through two fully connected layers and employs average pooling operations to perform channel-level weighted fusion. This fusion process generates a new feature map $Y_{\mathrm{IWS}}$ with enhanced feature representation, emphasizing important channels and suppressing less-relevant ones. Compared with the direct weighting of the input image and the anomaly map, the model can adaptively assign weights to different inputs to improve the segmentation performance of the model. The channel attention mechanism expression is as follows:

(16) $$Y_{\mathrm{IWS}}=\sigma \left(W_{2}\delta \left(W_{1}\mathrm{Avg}\left(X_{\mathrm{ori}}\right)+W_{1}\mathrm{Avg}\left(X_{\mathrm{ano}}\right)\right)\right),$$

where σ represents the sigmoid activation function, δ represents the ReLU activation function, Avg represents the average pooling operation, $X_{\mathrm{ori}}$ represents the synthetic defect image, $X_{\mathrm{ano}}$ represents the anomaly map, $W_{1}$ and $W_{2}$ are the weight matrices of two fully connected layers. The input images weighted by channel attention can be represented as follows:

(17) $$X_{ori,ano}=Concat\left(X_{ori},X_{ano}\right)⊙Y_{IWS},$$

**Figure 6:**
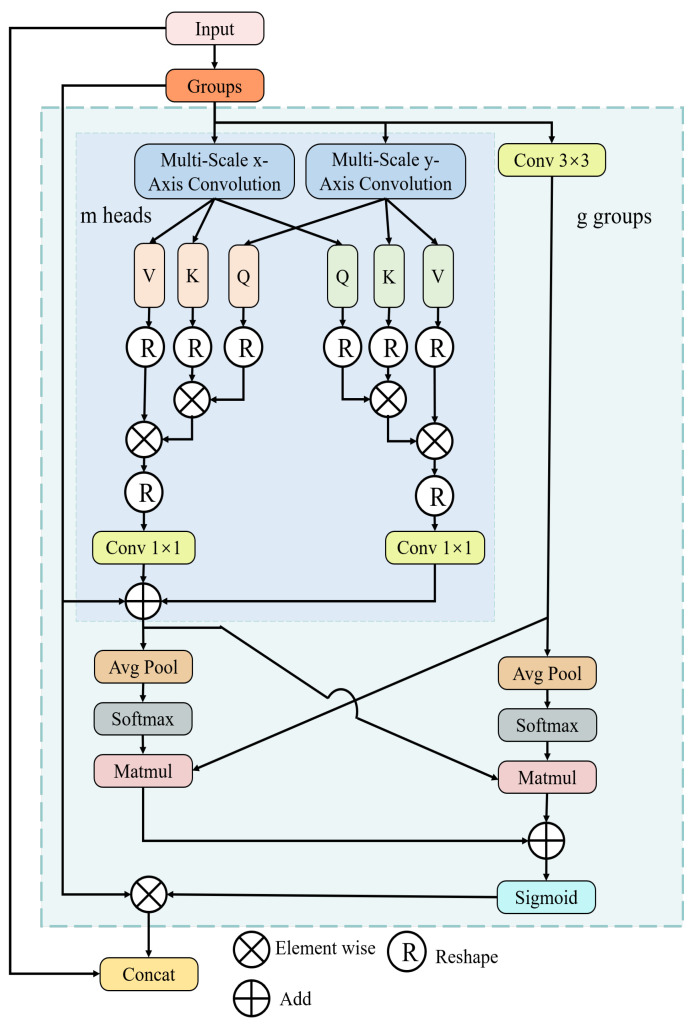
The architecture of multi-scale biaxial cross-attention.

**Figure 7:**
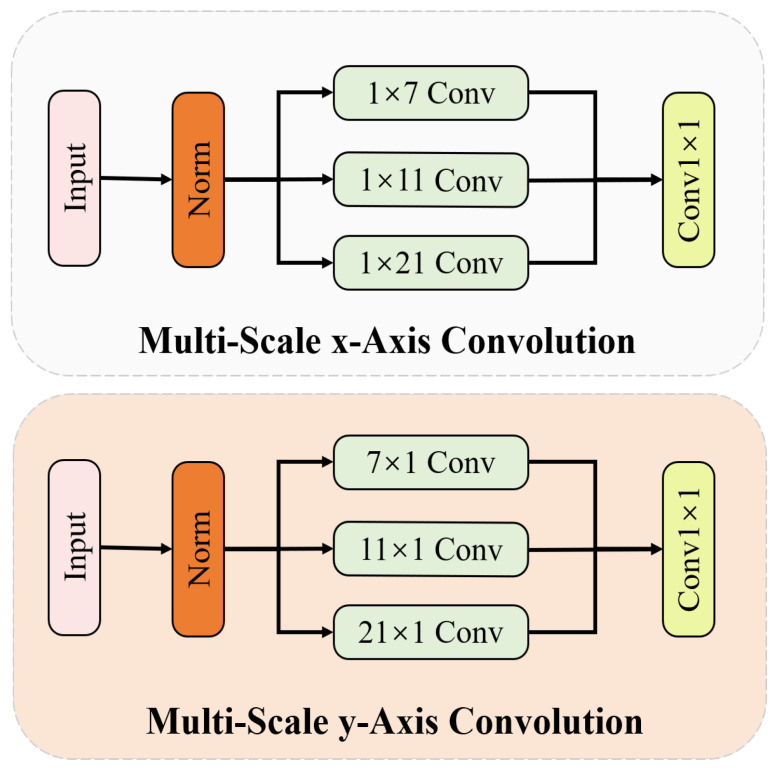
The architecture multi-scale axis convolution.

**Multi-scale biaxial cross-attention (MBCA)**: Inspired by EMA and MCA, we design a novel method named multi-scale biaxial cross-attention that integrates multi-scale features and axial features, aiming to better segment defect regions of various sizes and shapes. As illustrated in Figure 6, MBCA utilizes convolutional kernels of different sizes and shapes to capture local information from various defect regions as comprehensively as possible, thereby enhancing the model’s ability to represent defect features. The MBCA module divides the input feature map $X_{input}$ into *g* groups, and each set should pass through three branches. The specific feature extraction method of the multi-scale *x*-axis convolution and multi-scale *y*-axis convolution is illustrated in Figure 7. When performing feature extraction with axial cross-attention, each input feature map needs to undergo LayerNorm, followed by three strip convolutions of different shapes and then a 1×1 convolution operation. The formulation can be written as follows:

(18) $$F_{x}=Conv_{1\times 1}\left(\sum_{i=0}^{2}Conv1D_{i}\left(LayerNorm\left(X_{input}\right)\right)\right),$$

(19) $$F_{y}=Conv_{1\times 1}\left(\sum_{i=0}^{2}Conv1D_{i}\left(LayerNorm\left(X_{input}\right)\right)\right).$$

where $F_{x}$ and $F_{y}$ denote the output of multi-scale axis convolution and $\mathrm{Conv}1D_{i}$ is a 1D convolution operation. The specific kernel size is shown in Figure 7. Then, $F_{x}$ and $F_{y}$ are utilized to calculate the multi-head cross-attention between them. The calculation process can be written as follows:

(20) $$Att_{xy}=\mathrm{MA}\left(F_{y},F_{x}\right),$$

(21) $$Att_{yx}=\mathrm{MA}\left(F_{x},F_{y}\right).$$

MA is multi-head cross-attention. when $Att_{xy}$ and $Att_{yx}$ are obtained, the output of biaxis cross-attention is calculated as follows:

(22) $$F_{\mathrm{biaxial}}\;=Conv_{1\times 1}\left(Att_{xy}\right)+Conv_{1\times 1}\left(Att_{yx}\right)+X_{input}.$$

We represent the feature map obtained through the 3 × 3 convolutional kernel as $F_{3\times 3}$. We apply the AVG and Softmax operations to obtain their respective attention matrices as follows:

(23) $$Att_{\mathrm{biaxial}}\;=Softmax\left(AVG\left(F_{\mathrm{biaxial}}\right)\right),$$

(24) $$Att_{3\times 3\;}=Softmax\left(AVG\left(F_{3\times 3}\right)\right),$$

After that, we use cross multiplication to obtain their feature maps enhanced by the attention matrices, as shown in the following formula:

(25) $$F_{\mathrm{biaxial}}^{\prime }=Att_{3\times 3\;}⊙F_{\mathrm{biaxial}},$$

(26) $$F_{3\times 3\;}^{\prime }=Att_{\mathrm{biaxial}\;}⊙F_{3\times 3\;},$$

The feature map improved by MBCA attention can be computed through the following process:

(27) $$F_{MBCA}=\sigma \left(F_{\mathrm{biaxial}}^{\prime }⊕F_{3\times 3}^{\prime }\right)⊙X_{input.}$$

Since the reweighted matrix is calculated based on groups and there is no communication between groups, we aim to introduce global feature information. Therefore, we concatenate the input matrix with the objects to perform global feature fusion as follows:

(28) $$F_{MBCA}^{\prime }=Concat\left(F_{MBCA},X_{input}\right).$$

**Figure 8:**
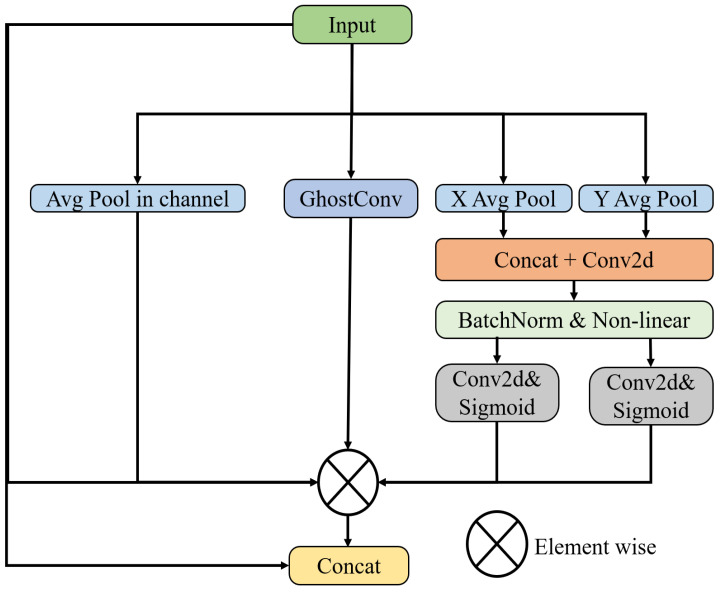
Framwork of CSA.

**Coordinate spatial attention (CSA):** After obtaining the feature map, based on coordinate attention and spatial attention, coordinate spatial attention is introduced to improve GalvaNet’s perception of coordinates and space. In this module, based on the spatial and coordinate attention mechanism, the model dynamically adjusts the weights of each position in the feature map regarding the spatial attention mechanism, allowing the model to focus more on important information (such as object boundaries) and texture [35]. Additionally, the coordinate attention focuses on the position information at different features through the computation of each position’s coordinate encoding to depict the feature map positions [36], thereby enhancing the model’s ability to utilize position information and improve its generalization capability. Therefore, our proposed attention fusion module assists the model in better focusing on spatial and positional information in the feature map, thereby enhancing and the segmentation performance of GalvaNet. The process of CSA is shown in Figure 8. First, we obtain the attention matrix of the feature map at the coordinate level:

(29) $$F_{\mathrm{concat}}\left(h_{\mathrm{cat}},w_{\mathrm{cat}}\right)=Concat\left(F_{\mathrm{avg}}\left(Y_{\mathrm{conv}}\right),F_{\max}\left(Y_{\mathrm{conv}}\right)\right),$$

(30) $$Att_{coor}=\sigma \left(Conv2d\left(F_{\mathrm{concat}}\left(h_{\mathrm{cat}},w_{\mathrm{cat}}\right)\right)\right).$$

In Formula (29), $F_{\max}$ and $F_{\mathrm{avg}}$ represent the max pooling and average pooling of the input feature maps, respectively. The Concat operation represents concatenating the obtained feature maps in the channel dimension. In Formula (30), after passing through the deep convolutional module, the feature map that focuses more on image position information obtained through coordinate attention is denoted as $Att_{\mathrm{coor}}$. This process involves computing the coordinate encoding for each position, such as row and column indices, to generate a unique weight matrix, where $h_{\mathrm{conv}},c_{\mathrm{conv}},w_{\mathrm{conv}}$ represent the height, width, and number of channels of the input feature map, respectively. Then, $Att_{\mathrm{spa}}$ represents the feature map with the enhanced spatial information obtained. CSA also uses AVG to compute the attention matrix of the input feature map at the spatial level to enhance the model’s ability to capture spatial features, as follows:

(31) $$Att_{spa}=\frac{1}{C_{conv}}\sum_{c=1}^{C_{conv}}Y_{\mathrm{conv}\;}\left(h_{\mathrm{conv}},w_{\mathrm{conv}},c_{\mathrm{conv}}\right).$$

We believe that the galvanized steel surfaces with complex textures have many overlapping features, so GhostConv [47] is introduced to obtain more non-redundant features $Y_{ghost}$ as shown in Formula (32):

(32) $$Y_{ghost}=GhostConv\left(Y_{\mathrm{conv}}\right).$$

Finally, we multiply the obtained attention matrix by the input feature map and stitch the multiplied matrix and the input feature map into channels to obtain the feature map $Y_{CSA}$. The feature map, enhanced by the coordinate spatial attention mechanism, is mapped back to a single channel as the segmentation mask output:

(33) $$Y_{CSA}=Concat\left(Y_{\mathrm{conv}},\left(Y_{\mathrm{conv}}⊙Att_{coor}⊙Att_{spa}⊙Y_{ghost}\right)\right).$$

The remaining network structure details of aggregated attention are shown in Table 1.

**Classification-aided module (CAM):** We aim for GalvaNet to have better interpretability and to generate more accurate anomaly maps, which is designed to improve the segmentation performance by incorporating a classification task. The fundamental requirement for a high-quality anomaly map is to ensure that its users recognize anomalies clearly, which in turn improves segmentation performance. The classification-aided module is shown in Table 2.

The feature processing and classification pipeline begins with the fusion of the single-channel mask output from the aggregated attention part and the 1024-channel feature map obtained from the deep convolutional modules. This fusion, conducted along the channel dimension, yields a comprehensive 1025-channel feature map. Subsequently, this fused feature map undergoes dimensionality reduction through convolutional processing, refining it into a more manageable 32-channel feature map. Next, both the 32-channel feature map and the single-channel mask go through global max pooling (GMP) operations and global average pooling (GAP) operations. These pooling operations are simultaneously utilized for both the features and the mask, yielding 64-dimensional feature representations for each. This parallel processing ensures that both global image features and segmentation-specific details are effectively captured and represented.

Simultaneously, the single-channel mask, representing segmentation details, utilizes the same pooling operations, generating a two-dimensional feature representation. These features encapsulate segmentation-specific information, contributing to a more comprehensive feature vector.

Following the pooling operations, the 64-dimensional feature representations from the feature map and the mask are concatenated with the 2-dimensional segmentation feature, resulting in a 66-dimensional feature vector. This concatenation effectively integrates information from different processing stages, combining both global image features and localized segmentation details. Finally, this 66-dimensional feature vector is input into a multi-layer perceptron (MLP) network for classification.

### 3.4. Loss Function

This loss [48] takes into account the loss of defect segmentation and the classification of defective images, placed at the end of the network for gradient-based descent algorithms, enabling simultaneous end-to-end learning. Unlike their approach [48], we did not prohibit the loss from the classification module from backpropagating to the segmentation module during loss propagation. The unified loss is defined as follows:

(34) $$L_{\mathrm{total}\;}=\lambda \cdot L_{\mathrm{seg}\;}+\left(1-\lambda \right)\cdot \phi \cdot L_{\mathrm{cls}}.$$

$L_{\mathrm{seg}}$ and $L_{\mathrm{cls}}$ represent the losses of segmentation and classification, respectively, utilizing cross-entropy loss. ϕ acts as an additional weight for classification loss, and λ balances each sub-network’s contribution to the final loss. It is important to note that λ and ϕ complement the learning rate η in stochastic gradient descent (SGD) rather than replacing it. By averaging the segmentation loss over all pixels, predominantly non-anomalous pixels, ϕ helps balance both losses, which may vary in scale.

To address the challenge of performing classification tasks on an anomaly map obtained from the aggregated attention part, we aim for a smooth transition in the balance factor associated with their respective loss functions. We express the transition process of this balancing factor as follows:

(35) $$\lambda =1-\frac{n}{2n_{\mathrm{epoch}}}.$$

In this context, *n* denotes the current epoch index and $n_{epoch}$ signifies the total number of training epochs. Failure to gradually balance the losses may result in gradient explosions during the learning process. This gradual transition in training emphasis from segmentation to classification is termed dynamically balanced loss.

## 4. Experiments and Results

### 4.1. Dataset

**Figure 9:**
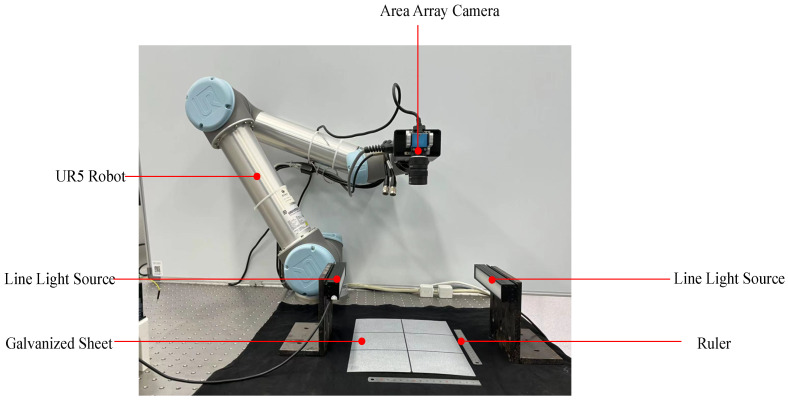
The surface defect collecting system for galvanized plates captures images of the galvanized plates using an area array camera operated by a UR5 robot under the illumination of an online light source.

**Figure 10:**
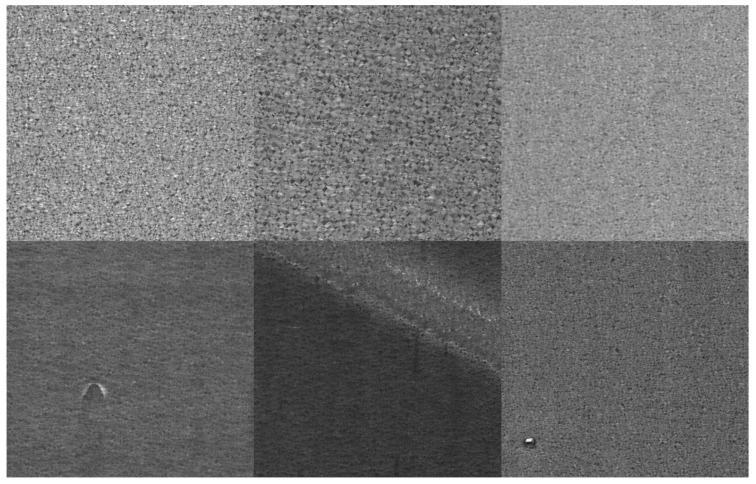
Spangle partial image visualization.

Applying unsupervised anomaly detection algorithms to detect defects on steel surfaces with complex textures is an emerging and critical task. However, there is currently no dataset that is specifically available for this task. Therefore, we utilized a UR5 robot to hold an industrial area scan camera produced by DAHENG IMAGING, version number MER-2000-19U3M, to capture high-resolution images of a galvanized sheet, ensuring that the distance between the camera and the sheet was kept constant. Additionally, we placed linear light sources on both sides and adjusted their brightness to simulate the light and shadow variations in the actual production environment. The specific system and equipment placement are shown in Figure 9. We captured te complex texture the galvanized sheet, known as spangle. The collected data were annotated at the pixel level by members of our team using ImageJ. We used the ImageJ’s smear pattern to mark the location of the defect. We named this collected and annotated dataset ’Spangles’. To our knowledge, this is the first visual image-based dataset for the detection of defects on galvanized sheet surfaces. We relied on our data acquisition system to collect 1521 images of galvanized sheets with varying texture densities. However, considering that some unsupervised defect detection methods tend to consume significant GPU and memory resources, we also aimed to complete the task with as few normal images as possible. We selected four different types of galvanized sheets with significant texture differences for our training set, with each type containing 25 images, totaling 100 images. The detailed information about this dataset is as follows:

**Spangles**: The dataset consists of 690 galvanized sheet images with a resolution of 1024 × 1024. Among these, 297 images exhibit defects and 393 images are defect-free. Approximately 43.04% of the images are anomalous. The training set includes only 100 defect-free images, with 25 images from each level, which are classified into four levels based on the spangle density of the galvanized sheets, from largest to smallest, as levels 1 to 4. The remaining images are included in the test set. Based on the empirical classification, among the defect images, 200 images have tiny defects (small-area cat whiskers and small zinc dross) and 97 images have major defects (notch marks, horizontal stripes, zinc ash imprint oil spots, large-area cat whiskers, and large zinc dross). Examples of the dataset are shown in Figure 10.

To further investigate the generalization of our method, we also utilized the Kolektor Surface-Defect Dataset 2 (KSDD2) [48] to evaluate the effectiveness of our algorithm.

### 4.2. Experimental Configuration

This algorithm is implemented using a device with an Ubuntu 22.04 OS, Intel 12700K CPU, NVidia GeForce RTX 4090 graphic card, and 64 GB of memory, as shown in Table 3. Apart from replicating the Memseg, PNI, and SimpleNet directly in the official source code, all other comparative experiments were implemented using Anomalib [49].

For ViT-Query, the feature aggregation parameter *r* is {1,3,5,7}. For GalvaNet, the training hyperparameters for the network are as follows: a dilation convolutional kernel size of 7 trained with SGD, with no momentum and with no weight decay. Regarding the training of the network, we employ an automatic adjustment mechanism for the weights of segmentation loss and classification loss. These weights are automatically adjusted every epoch, starting with a segmentation loss weight of 1.0 and a classification loss weight of 0. The loss function used for training is BCEWithLogitsLoss [50].

To assess the performance of the methods, we adopted the area under the receiver operator curve (AUROC) and the F1-score as metrics. The AUC value represents the area under the ROC curve. By applying different thresholds, multiple sets of coordinates can be obtained and calculated, as shown in Equation (38). Here, TN denotes the number of samples correctly classified as negative cases, and FN denotes the number of samples incorrectly classified as negative cases. This evaluation metric effectively measures the model’s ability to recognize positive samples [51] and can be computed as follows:

(36) $$\mathrm{TPR}\;\left(\mathrm{Recall}\right)=\frac{TP}{TP+FN},$$

(37) $$\mathrm{FPR}=\frac{FP}{TN+FP},$$

(38) $$\mathrm{AUROC}=\int_{0}^{1}TPR\left({\mathrm{FPR}}^{-1}\left(x\right)\right)\;dx,$$

(39) $$\mathrm{Precision}=\frac{TP}{TP+FP},$$

(40) $$F1=\frac{2*Recall*Precision}{Recall+Precision}.$$

### 4.3. Results on Spangles

The experiment results of anomaly detection in spangles are presented in Table 4. We compared our proposed GalvaNet algorithm with several conventional algorithms (DRÆM, reverse distillation (RD) [52], Padim [53], CFlow, FastFlow [54], PatchCore, PNI, SimpleNet, Memseg) in terms of image-level AUROC (I-AUROC), image-level F1-score (I-F1), pixel-level AUROC (P-AUROC), and pixel-level F1-score (P-F1). It can be observed that the majority of the unsupervised anomaly detection algorithms demonstrated satisfactory performance at the image level. The proposed VQGNet algorithm demonstrated an anomaly detection performance of 99.6% I-AUROC, 98.8% I-F1, and an anomaly localization performance of 97.0% P-AUROC and 80.3 P-F1, surpassing PNI by 0.1% and 0.3%, respectively, at the image level. Although FastFlow, based on a powerful transformer, and SimpleNet, combining representation and generative methods, also show superior performance, our proposed algorithm outperforms them. Additionally, at the pixel level, the P-AUROC and P-F1 are improved by 6.2% and 51.7% over the second-best SimpleNet and Patchcore, respectively. The seemingly counterintuitive changes in I-AUROC and the F1-score are due to the focus on detecting small defects in galvanized sheets. The dataset contains a large number of small defects, leading to variations in P-AUROC and P-F1 across the different algorithms.

**Figure 11:**
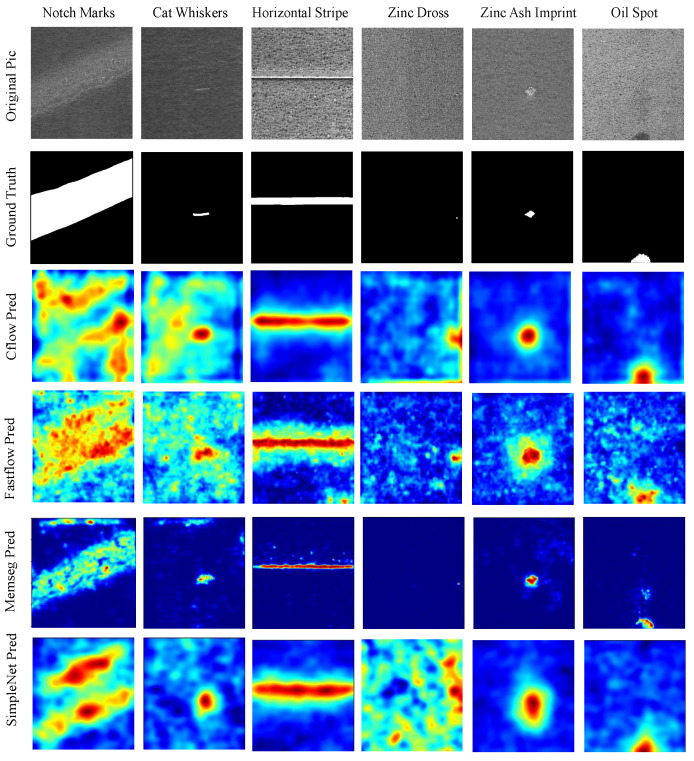
Visualization of defect segmentation results on spangles. PatchCore, PNI, and VQGNet’s visual results are presented in Figure 13.

**Figure 12:**
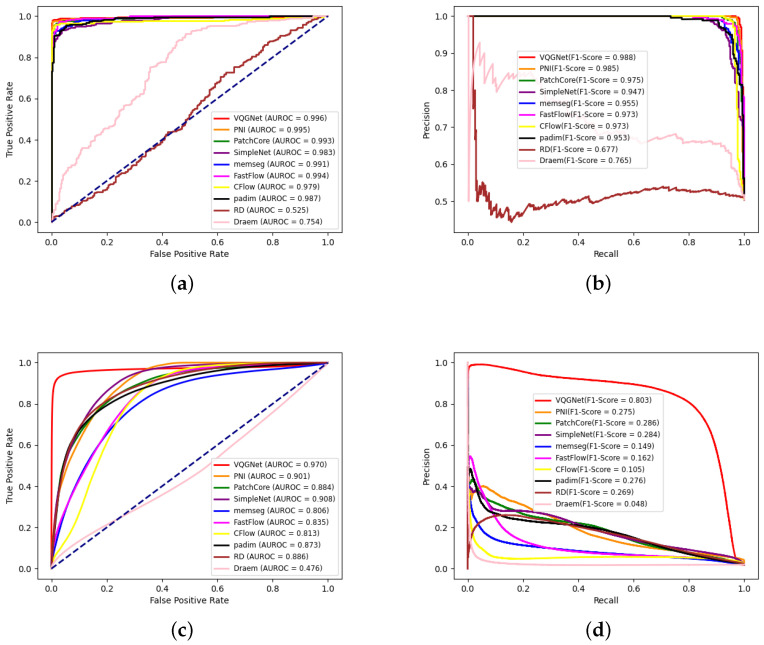
ROC and precision–recall curves at the image level and pixel level of all algorithms on spangles. (**a**) Image-level ROC curves; (**b**) image-level precision–recall curves; (**c**) pixel-level ROC curves; (**d**) pixel-level precision–recall curves.

**Figure 13:**
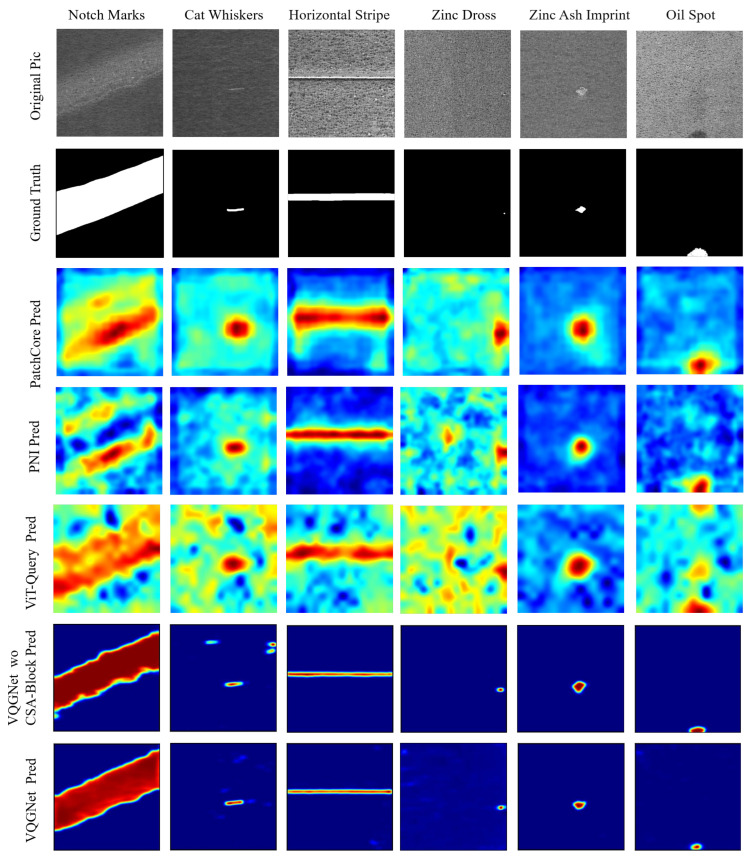
Visualization of defect segmentation results on spangles.

Nonetheless, the improvements in P-AUROC and P-F1 are still remarkable. We believe this is because the synthetic defect images used in network training effectively handled various complex defects occurring in real texture images, learning more precise localization information. As shown in Figure 11, Figure 12 and Figure 13, compared to the visual results of the other algorithms’ anomaly maps, the visual results of VQGNet are significantly better. The visual results of VQGNet are mostly within the key areas indicated by ViT-Query’s visualization, specifically the red regions. This suggests that ViT-Query’s anomaly map provides sufficient prior knowledge of GalvaNet. ViT-Query tends to focus more on global information in the task of anomaly detection on galvanized surfaces, while its attention to local information is relatively weaker. This explains why, despite ViT-Query’s high classification metrics, its segmentation metrics are less satisfactory. However, it is reassuring that ViT-Query provides a rough range for the segmentation results, within which we can perform a more detailed search. ViT-Query exhibits good performance in certain areas with significant defects, such as notch marks, and it better indicates the locations of defects compared to the other algorithms. Leveraging the enhanced defect recognition capability gained from normal images and synthetic defect images, GalvaNet produces a superior Anomaly Map, even though VQGNet may lose some information.

We also draw ROC and precision–recall curves at the image level and pixel level of all methods, as shown in Figure 12. On the image level, VQGNet, PNI, and PatchCore achieve a similar performance, and VQGNet is slightly better than PNI and PatchCore. On the pixel-level, the AUC of VQGNet is much larger than that of other methods. Based on the precision–recall curves, VQGNet reaches a higher precision than that of other methods with the same recalls.

### 4.4. Comparative Experiments on KSDD2

**Figure 14:**
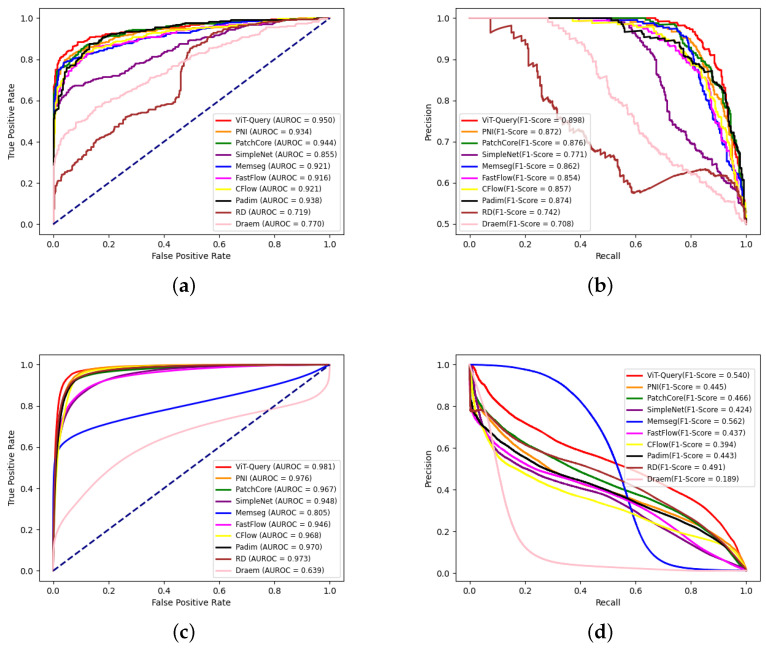
ROC and precision–recall curve at the image and pixel levels of all algorithms on KSDD2. (**a**) Image-level ROC curves; (**b**) image-level precision–recall curves; (**c**) pixel-level ROC curves; (**d**) pixel-level precision–recall curves.

**Figure 15:**
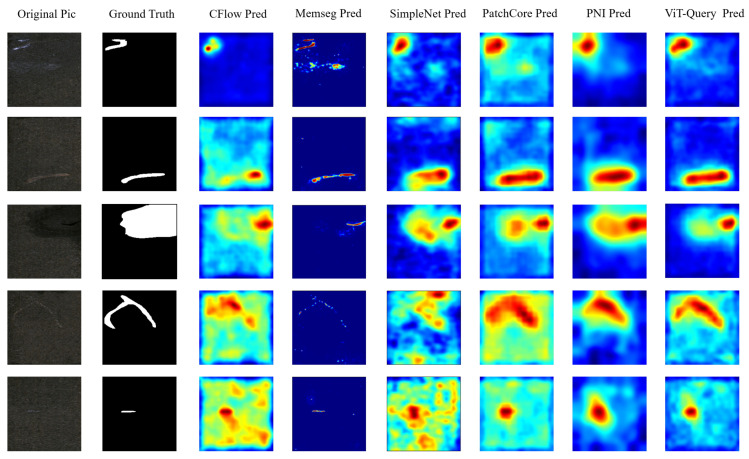
Visualization of defect segmentation results on KSDD2.

To explore the perceptual encoding capability of ViT-Query for RGB images, we conducted comparative experiments on the KSDD2 dataset. To balance the normal and abnormal samples in the dataset, we selected 356 images in the training set and 356 images with and without defects as the test set. Under this experimental setup, we conducted unsupervised defect detection. Table 5 shows that our model achieved state-of-the-art results, but our method ranked second in terms of P-F1 score. Compared to PatchCore, ViT-Query improved by 0.6% in I-AUROC and 2.1% in I-F1. Additionally, compared to PNI, our method improved by 0.5% in P-AUROC. We also draw ROC and precision–recall curves at the image-level and pixel-level of all the methods, as shown in Figure 14. Figure 14 clearly shows that our method has a certain advantage over the other algorithms. We plotted the visualized results of the experiments in Figure 15. Our algorithm demonstrates superior segmentation performance for various types of defects. It is apparent that the ViT-based ViT-Query more accurately delineates defect shapes compared to the ResNet-based PatchCore and PNI. Moreover, our method demonstrates better generalization compared to MemSeg’s over-segmentation.

### 4.5. Ablation Study

**Figure 16:**
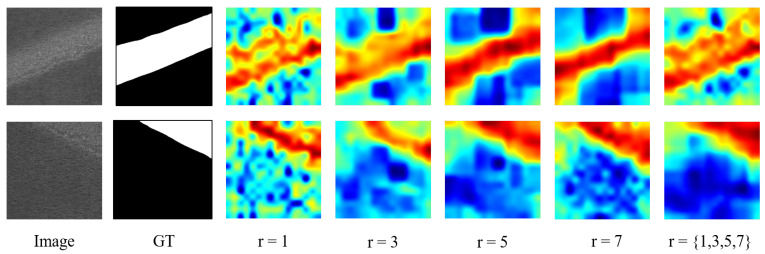
Visualization of the aggregation of different kernel sizes.

**Effects of the neighbor feature aggregation parameter *r***: As shown in the Table 6, choosing different sizes of kernels for neighborhood feature aggregation significantly affects the performance of ViT-Query. The experimental results indicate that with r={1,3,5,7}, ViT-Query achieves better comprehensive results in I-AUROC, I-F1, P-AUROC, and P-F1 compared to other settings. During the experimental process, we observed that using small kernels for neighbor feature aggregation is more sensitive to small-scale features and the boundaries within the image (i.e., the boundary between defect areas and normal areas). However, due to the texture of the steel surface, this approach is prone to misjudgment. On the other hand, larger kernels tend to capture defect features over a larger area, but this leads to poorer segmentation performance at the boundary between defect areas and normal areas, as shown in Figure 16.

**Ablation study on modules of GalvaNet**: We conducted an ablation study to verify the effects of three components in our proposed GalvaNet algorithm, IWS, MBCA, and CSA. The results are presented in Table 7. The following observations can be made:

1. The baseline model, which excludes these three components, is essentially equivalent to ViT-Query.
2. Integrating high-resolution image information with the IWS to train GalvaNet without CSA and MBCA significantly increases P-F1 from 44.7% to 71.8%. However, simply adding attention mechanisms to channels for weighted integration can cause global information to be biased towards specific channels, thereby affecting the global information in the anomaly map. This resulted in a decline in the classification performance, with I-AUROC and I-F1 dropping from 99.4% and 98.4% to 95.1% and 85.1%, respectively. When we turn our attention to the visualized anomaly map in Figure 13, we make the following observations: When we do not add the CSA and MBCA, the remaining model (GalvaNet without CSA, MBCA) relies more on the anomaly map provided by ViT-Query. Based on the visualization results of the cat whiskers, we can observe that because the channel attention performs weighted operations across the entire channel, the remaining model gives high attention to some secondary focus areas provided by ViT-Query’s anomaly map.
3. Introducing the CSA after the IWS to focus on the spatial and coordinate information of the original high-resolution images and the anomaly maps allowed us to better integrate global and local features. This approach aimed to maintain classification performance while also improving segmentation tasks. Segmentation performance saw substantial improvements, with P-AUROC and P-F1 rising from 91.2% and 71.8% to 96.0% and 75.0%, respectively. By introducing CSA, the model can better learn the rich semantic information within the anomaly map and balance it with the defect segmentation capability obtained from normal images and synthetic defect images. Thus, after adding CSA, the shape of the defect areas is more similar to the ground truth, and the anomaly scores in the normal areas are lower, as shown in Figure 13.
4. When we introduced MBCA to VQGNet, which already includes IWS and CSA, we found that the model’s image-level metrics surpassed those of ViT-Query, with improvements of 0.2% in I-AUROC and 0.4% in I-F1. Additionally, at the pixel level, P-AUROC and P-F1 improved by 1% and 5.3%, respectively.
5. Overall, these modules collectively enhance the model’s ability to classify and segment anomalies at both the image level and the pixel level. After incorporating all the proposed modules, a significant improvement in the model is observed: I-AUROC and I-F1 improved by 0.2% and 0.4%, respectively, while P-AUROC and P-F1 improved by 2.7% and 35.6%, respectively.

## 5. Conclusions

In this paper, we propose VQGNet, an unsupervised anomaly detection method that can precisely recognize and segment defects in complexly textured steel simultaneously. GalvaNet, as a main component of VQGNet, comprises IWS, MBCA, CSA, and CAM. It detects and segments defects by integrating various features from both the original image and the anomaly map. IWS dynamically weights the input anomaly map and original image, MBCA enhances the ability to select multi-scale features, and CSA strengthens the model’s ability to extract the coordinate and spatial information of features. The refined anomaly maps and fused features are both input into the CAM for the final classification and segmentation. ViT-Query is proposed to generate the anomaly maps more confidently via multi-scale feature fusion and neighbor feature aggregation. Moreover, an anomaly generation method suitable for grayscale images is introduced to facilitate the model’s learning on the anomalous samples. A series of experiments is carried out using real, complexly textured steel images. VQGNet achieves state-of-the-art (SOTA) performance on the collected spangle dataset, with an I-AUROC of 99.6%, I-F1 of 98.8%, P-AUROC of 97.0%, and P-F1 of 80.3%. Moreover, ViT-Query shows that ViT-Query achieved a P-AUROC of 94.3% and a P-F1 of 44.7% on the spangles dataset and state-of-the-art performance on KSDD2, which demonstrates that compared to other unsupervised anomaly detection algorithms, ViT-Query can obtain anomaly maps with a higher reliability. Furthermore, the ablation studies further confirmed the contributions of the IWS, MBCA, and CSA components in GalvaNet, highlighting their roles in enhancing defect segmentation and maintaining a high classification accuracy. The visual examples of the anomaly generator demonstrate that our generated defects bear a significant similarity to real defects.

The limitations of our study are as follows. In this work, we did not integrate GalvaNet with every algorithm due to the observed variation in the distribution ranges of the anomaly maps produced using different methods. Directly normalizing these maps would affect the anomaly scores of certain anomalous images, thereby compromising the fairness and effectiveness of the analysis. Consequently, GalvaNet was not employed as a universal module; rather, it was specifically combined with ViT-Query to develop VQGNet. Additionally, our empirical results indicate that while VQGNet enhances predictive performance, this improvement comes at the expense of increased computational resource usage and reduced inference speed.

Overall, VQGNet showcases a promising approach for accurate and efficient anomaly detection and segmentation in complexly textured steel, offering valuable insights for future research and applications in this domain.

In the future, we hope to further improve the segmentation effect of the algorithm on panels with complex textures and improve our algorithm for use on other kinds of materials with complex textures.

## Figures and Tables

**Table 1:** Architecture details for aggregated attention.

Layer	Kernel Size	Feature
Input image	–	2
IWS	–	2
2 × Conv2D	5×5	32
Max-pool	2×2	32
3 × Conv2D	5×5	64
MBCA	–	64
Max-pool	2×2	64
4 × Conv2D	5×5	64
Max-pool	2×2	64
Conv2D	5×5	1024
CSA	–	1024
2 × Conv2D	5×5	1024

**Table 2:** Architecture details for classification-aided module.

Layer	Kernel Size	Feature
Segmentation mask	–	1
Fusion image	–	1025
Max-pool	2×2	1025
Conv2D	5×5	8
Max-pool	2×2	8
Conv2D	5×5	16
Max-pool	2×2	16
Conv2D	5×5	32
GMP	–	32
GAP	–	32
GMP (seg mask)	–	1
GAP (seg mask)	–	1
Concat feature	–	66
Classification output	–	1

**Table 3:** Experiment environment parameters.

Hardware or Software	Version
CPU	Intel 12700K
GPU	RTX 4090
Operating system	Ubuntu 22.04
CUDA version	11.8
Driver version	546.80
Python	3.8

**Table 4:** Comparison results on spangles. Anomaly detection and localization performance are measured based on I-AUROC [%], I-F1 [%], P-AUROC [%], and P-F1 [%], respectively.

Models	Size	I-AUROC	I-F1	P-AUROC	P-F1
DRÆM	224	75.4	76.5	47.6	4.8
RD	224	52.5	67.7	88.6	26.9
Padim	224	98.7	95.3	87.3	27.6
CFlow	224	97.9	97.3	81.3	10.5
FastFlow	224	99.4	97.3	83.5	16.2
Memseg	224	99.1	95.5	80.6	14.9
SimpleNet	384	98.3	94.7	90.8	28.4
PatchCore	224	99.3	97.5	88.4	28.6
PNI	224	99.5	98.5	90.1	27.5
**VQGNet**	224, 1024	**99.6**	**98.8**	**97.0**	**80.3**

**Table 5:** Comparison results on KolektorSDD2. Anomaly detection and localization performance are measured based I-AUROC [%], I-F1 [%], P-AUROC [%], and P-F1 [%], respectively.

Models	I-AUROC	I-F1	P-AUROC	P-F1
DRÆM	77.0	70.8	63.9	18.9
RD	71.9	74.2	97.3	49.1
Padim	93.8	87.4	97.0	44.3
CFlow	92.1	85.7	96.8	39.4
FastFlow	91.6	85.4	94.6	43.7
Memseg	92.1	86.2	80.5	**56.2**
SimpleNet	85.5	77.1	94.8	42.4
PatchCore	94.4	87.6	96.7	44.6
PNI	93.4	87.2	97.6	44.5
**ViT-Query**	**95.0**	**89.7**	**98.1**	54.0

**Table 6:** Ablation study of kernel aggregation results based on size of spangles.

Model	I-AUROC	I-F1	P-AUROC	P-F1
ViT-Q (1)	98.6	97.9	91.9	38.3
ViT-Q (3)	98.7	98.0	92.1	38.5
ViT-Q (5)	98.9	97.6	92.0	38.3
ViT-Q (7)	98.4	97.5	92.2	39.5
ViT-Q (1, 3)	99.3	98.1	92.0	39.7
ViT-Q (1, 3, 5)	99.4	98.1	93.5	42.8
ViT-Q (1, 3, 5, 7)	**99.4**	**98.4**	**94.3**	**44.7**

**Table 7:** The ablation study results of the module of GalvaNet on CASE spangles. Anomaly detection and localization performance are measured in I-AUROC [%], I-F1 [%], P-AUROC [%], and P-F1 [%], respectively.

Model	IWS	MBCA	CSA	I-AUROC	I-F1	P-AUROC	P-F1
ViT-Query				99.4	98.4	94.3	44.7
VQGNet w/o CSA, MBCA	✓			95.1	85.1	91.2	71.8
VQGNet w/o CSA	✓	✓		99.5	98.3	94.3	72.7
VQGNet w/o MBCA	✓		✓	98.5	96.8	96.0	75.0
VQGNet w/o IWS		✓	✓	99.2	97.7	96.2	79.7
**VQGNet**	✓	✓	✓	**99.6**	**98.8**	**97.0**	**80.3**

## Data Availability

Data and code will be available upon request.
